# Exploring the perceptions of sedentary behaviour in community-dwelling older adults aged 75 and older: a series of focus group interviews

**DOI:** 10.1186/s12889-025-23793-y

**Published:** 2025-07-26

**Authors:** Ragy Tadrous, Anne Forster, Amanda Farrin, Peter A. Coventry, Andrew Clegg

**Affiliations:** 1 University of Leeds, Leeds, UK; 2 University of York, York, UK; 3 Academic Unit for Ageing and Stroke Research, Temple Bank House, Bradford Royal Infirmary Duckworth Lane, Bradford, BD9 6RJ UK

**Keywords:** Frailty, Sedentary behaviour, Physical inactivity, Focus groups, Older adults, Qualitative

## Abstract

**Background:**

Older adults are the most sedentary and fastest-growing demographic, yet adults aged ≥ 75 years are underrepresented in sedentary behaviour research. This study qualitatively explored how this age group perceives sedentary behaviour, the activities they perform in sitting and standing, and the barriers and facilitators to reducing their sedentary behaviour.

**Methods:**

Four focus groups were conducted with a consistent group of 6 community-dwelling older adults aged ≥ 75 years from West Yorkshire were held between October-December 2022. Audio recordings and focus group notes were transcribed verbatim and an inductive and deductive thematic analysis was conducted. The activities performed in sitting and standing were charted to the ecological model of sedentary behaviour, and barriers and facilitators to reducing sedentary time were charted to the Capability Opportunity Motivation-Behaviour (COM-B) framework.

**Results:**

Participants were largely unaware of their sedentary behaviour or the associated health risks. Sitting activities were predominantly leisurely in nature, and occurred in older adults’ homes. Barriers and facilitators to reducing sedentary behaviour were mapped to the COM-B model. Key influences included physical and mental health, environmental constraints, social support, ingrained routines, and limited awareness of the health impacts of prolonged sitting. Analytical themes included the perceived progression of sedentary behaviour throughout older adulthood; the impact of prolonged sitting on sleep; and the role of social connectedness in reducing sedentary time.

**Conclusions:**

This study provided insights into older adults’ reports of sedentary behaviour progressing throughout older adulthood. When compared to the wider literature, sedentary behaviour in adults aged ≥ 75 years present similarly to a younger subset of older adults with regards to the activities performed in sitting, and the barriers and facilitators to reducing their sedentary time. However, the activities performed in sitting may be performed for longer, and the barriers to reducing sedentary behaviour may present more frequently. Social support appears valuable when attempting to reduce sedentary time, however, further research is necessary to explore the views of older adults who are socially isolated.

**Supplementary Information:**

The online version contains supplementary material available at 10.1186/s12889-025-23793-y.

## Introduction

Sedentary behaviour is defined as “any waking behaviour characterized by an energy expenditure ≤ 1.5 METs while in a sitting or reclining posture” [1]. Sedentary behaviour can be distinguished from physical inactivity, which is defined as not meeting the recommended levels of moderate-vigorous intensity physical activity [1]. Sedentary behaviour is associated with adverse health outcomes including poor self-reported health, decreased physical function, and higher healthcare usage [2]. Older adults are the fastest-growing demographic globally; and with approximately 67% of older adults spending more than 8.5 h per day being sedentary, they are also the most sedentary group in society [3, 4]. As such, the importance of reducing sedentary behaviour in older adults has been highlighted by the World Health Organisation [5].

A review by Chastin et al. [6] suggests that interventions to reduce sedentary behaviour in community-dwelling older adults may reduce sedentary time (− 44.91 min/day, 95% CI − 93.13 to 3.32 min). Additionally, a thematic synthesis conducted by Compernolle et al. [7] detailed the challenges associated with reducing sedentary behaviour in older adults. However, the studies which inform these reviews were predominantly informed by a younger subset of older adults, with only one of seven randomised controlled studies [8] and three of 15 qualitative articles [9–11] included in these reviews conducted with adults aged above 75. This underrepresentation of adults ≥ 75 years in sedentary behaviour research is pressing, as this population is expected to double in the next 30 years [12].

The health status of older adults Is also expected to change, with the percentage of adults aged 75–84 experiencing multi-morbidity predicted to increase from 61.9% in 2015 to 75.9% by 2035 [13]. The incidence and severity of frailty have also increased with age in recent years [14]. Age-related physical decline has also raised global concerns due to its association with higher rates of falls, hospitalisations, disability, and loss of independence [15]. Since sedentary behaviour is linked to increased frailty [2], reducing sitting time may be a viable strategy for managing frailty in older adults. Targeted approaches to reducing sedentary behaviour in adults aged ≥ 75 years may be warranted to account for the increasing health needs and the varying levels of sedentarism, frailty, perceptions and care needs present [13, 16]. This study aimed to qualitatively profile the sedentary behaviour of older adults aged ≥ 75 years. The objectives included:

i. Ascertaining older adults’ understanding of sedentary behaviour.
ii. Identifying the activities performed in sitting and standing.
iii. Charting the barriers and facilitators to reducing sedentary time.
iv. Exploring older adults’ perspectives on sedentary behaviour throughout older adulthood.

## Methods

Four semi-structured focus groups were conducted between September and December 2022 in the Bradford Institute for Health Research (BIHR) with adults aged ≥ 75 years to co-produce an intervention to reduce sedentary behaviour in community-dwelling older adults [17]. The findings reported in this study predominantly emerged from the first two focus groups which focused on participants’ understanding of sedentary behaviour, the activities they perform in sitting and standing, and the barriers and facilitators to reducing their sedentary behaviour. Each focus group lasted two hours and was audio-recorded, transcribed verbatim and thematically analysed.

Focus groups are recommended to help mitigate potential power differences between participants and researchers, which is vital for shared decision-making necessary for co-production [18]. This study deviates from traditional focus group methodology, which typically involves multiple independent groups, a decision that was made to support the co-production process. Maintaining a consistent group of participants fostered familiarity, continuity, and deeper engagement with the iterative development of the intervention. Ethical approval was obtained from the University of Leeds School of Medicine Research Ethics Committee (SoMREC project number MREC-21-052).

### Sampling and focus group size

Although the initial plan was to recruit older adults aged ≥ 75 years and their informal carers, most participants reported not receiving informal care, so pragmatically only adults were included. Purposive sampling considered age, living arrangements, and sedentariness to recruit a diverse group of older adults. Frailty classification, measured by the Electronic Frailty Index (eFI), was used to account for heterogeneity in health status, with higher eFI scores correlating with greater healthcare needs [19]. Higher frailty severity as measured by eFI has been shown to correlate with higher community healthcare requirements [20]. No consensus has been reached regarding the optimal number of participants in focus groups; however, the size of each group should be sufficient to foster debate but not so large that it inhibits participants from sharing their views. Recruiting 5–10 individuals to account for cancellations has been recommended [21]. As such, we sought to recruit between 5 and 7 older adults.

### Recruitment

Participants were recruited from the Community Ageing Research 75 + Study (CARE75+) cohort. The CARE75 + cohort is a longitudinal cohort study of community-dwelling older adults. The cohort consists of over 1000 older adults aged 75 or above, of which participants optionally consent to be contacted about future research. The CARE75 + study is led by the Academic Unit for Ageing and Stroke Research (ASR), University of Leeds, based at the BIHR, Bradford Teaching Hospitals NHS Foundation Trust. CARE75 + is funded by the National Institute for Health and Care Research, Yorkshire and Humber Applied Research Collaborations NIHR200166 [22, 23].

The recruitment process took place over three weeks. The lead author worked with the CARE75 + cohort manager to identify potential participants based on the eligibility criteria. Eligible individuals were aged ≥ 75 years, community-dwelling (i.e., living at home and not in residential or nursing homes), and living in either Leeds or Bradford to minimise travel burden to the BIHR. Additional inclusion criteria included the ability to stand and ambulate independently, provide written informed consent, and communicate in English. Participants were required to have an electronic Frailty Index (eFI) score indicating mild (0.13–0.24), moderate (0.25–0.36), or severe frailty (> 0.36), and be available to attend at least three of the four planned focus group sessions.

Once identified, the lead author sent potential participants an advertisement for the study and a participant information leaflet. After seven days, potential participants were contacted by phone by the lead author, provided with information about the study, and could ask any study-related questions. If a desire to participate in the study was expressed, verbal consent was sought over the telephone, and written informed consent was obtained during the first focus group. Recruited participants provided demographic data, with frailty measures (eFI) from the CARE75 + cohort. The timing of the most recent eFI update varied across participants, with some having scores updated within the last 12 months, and others having scores last updated more than 12 months before the first focus group meeting. Up-to-date information about sedentary time was provided through the MOST questionnaire at study onset.

### Topic guides

Semi-structured topic guides (Supplemental) were created in anticipation of each focus group and were informed by a preceding mixed-method review and discussions with researchers experienced in conducting focus groups about sedentary behaviour [24]. Additionally, the topic guides served a dual purpose as they mapped to the stages of the Behaviour Change Wheel and Theoretical Domains Framework and helped inform the intervention development that will be reported elsewhere. The focus groups followed an iterative approach, with the discussions in earlier sessions informing subsequent sessions. The first focus group explored the groups’ understanding of sedentary behaviour and the activities performed in sitting and standing, and the second focus group explored the behavioural determinants of sedentary behaviour in this population. The third and fourth focus groups focused on identifying and refining strategies to reduce sedentary behaviour. As such, the data presented in this qualitative analysis predominantly emerged from the first and second focus groups.

### Data collection

Each of the four focus groups lasted approximately two hours, and aligned to stages of the Behaviour Change Wheel [17]. Data were gathered through audio recordings, worksheet activities, and field notes. Audio recordings were used to accurately capture the content of the discussion and inform the thematic analysis. All group discussions were audio-recorded and transcribed verbatim by the lead author (RT), but discussions between participants during breakout activities were not. Instead, participants would provide summaries of what they discussed to the group at the end of each activity that were recorded and analysed. Worksheets were also used to capture additional points, including non-verbalised responses (e.g., activities performed in sitting that were not read aloud). A facilitator (SAH/SK) took field notes during each focus group that were then transcribed and analysed. To ensure the accuracy of the field notes, the facilitators provided a verbal summary of the main discussions at the end of each focus group and participants provided their input on if the summary accurately reflected the discussions. The facilitators debriefed after each session to discuss key points. Summaries of earlier focus groups were provided during each focus group.

Data collection and analysis took place iteratively: after each focus group, data were analysed and used to inform the content of subsequent focus groups. This iterative process enabled emerging ideas, themes, and uncertainties to be revisited, refined, or explored in greater detail in later sessions. The consistent nature of the focus groups, and the familiarity between group members, enabled continuity of discussions and allowed for deeper engagement regarding key topics. The final thematic framework reflected the cumulative insights across sessions and the evolving nature of participants’ reflections.

### Data analysis

Reflexive thematic analysis (RTA) is an approach to analysing qualitative data and is described as a “method for identifying, analysing and reporting patterns (themes) within data” [25]. It also allows for a broader understanding of a problem [26], and can be used to determine the components that influence an issue generated by participants [27]. RTA is a flexible approach that can identify themes inductively, deductively or through a mixed approach [28]. For these reasons, RTA was used to analyse qualitative data obtained from the focus groups. An inductive and deductive thematic analysis was conducted using NVivo guided by the methodology described by Braun and Clarke [25]. A deductive approach was used to chart the activities performed in sitting and standing to the ecological model of sedentary behaviour into the following contexts: leisure time, transport, household, and occupation [29].

As these findings emerged from a co-production process to develop an intervention to reduce sedentary behaviour that was guided by the Behaviour Change Wheel [17], we charted barriers and facilitators reported by participants to the relevant subdomains of the Capability, Opportunity, Motivation – Behaviour (COM-B) model of behaviour [30]. This was completed as part of the behavioural diagnosis to understand the behavioural determinants that underpinned sedentary behaviour in this sample. Capability refers to an individual’s physical and psychological capacity to reduce their sedentary behaviour; opportunity refers to the external physical and social factors that enable or prompt a behaviour, and motivation refers to reflective (such as conscious planning and decision-making) and automatic processes (such as habits and emotional responses) that influence behaviour [30].

One individual (R.T.) completed the analysis, with regular input from the other authors. This process consisted of six steps:

- Data Transcription: Responses were audio-recorded and transcribed verbatim by the study assessor (R.T). Data was then read, re-read and initial ideas from the responses were noted.
- Generating Initial Themes: Interesting features of the data were coded using a systematic approach across the complete data set. Interesting aspects from the responses were also noted.
- Searching for Themes:Similar codes that were generated were collated into potential themes. All relevant data were grouped under these specific themes.
- Reviewing Themes: Themes were reviewed in relation to the identified codes and the entire data set. In doing so, this process generated a thematic ‘map’ of the analysed data.
- Defining and Naming Themes:Themes were continuously analysed and refined based on specific aspects identified to create an overall story of the analysed data. Clear definitions and names were created for each theme.
- Producing the Report:Vivid and compelling extracts from responses were selected that related to the specific research questions and literature to produce a comprehensive report of the analysis

### Research team and reflexivity

The lead author is a male doctoral researcher with a background in Physiotherapy. He is experienced in qualitative research and underwent training in qualitative and participatory research methods and facilitated the focus groups. The research team included four supervisors, from a variety of disciplines with extensive research experience with older adults, intervention development, health psychology and behaviour change. The author group is gender balanced and consist**s** of junior and senior researchers from a variety of disciplines, with some authors being from marginalised groups. No members of the research team had any relationship with the participants prior to study commencement. Participants were aware that the focus groups presented in this study were a part of the lead authors’ doctoral work and were being used to inform the development of an intervention to reduce sedentary behaviour in community-dwelling older adults.

## Results

### Participant characteristics

The group comprised six older adults and two researchers (RT and SAH/SK). A total of 23 older adults met the eligibility criteria. The most frequently cited reasons for refusing to participate included a lack of interest (*n* = 9), other commitments (*n* = 5), and transport difficulties (*n* = 4). The demographic characteristics of the recruited participants are provided in Table 1. Participants had an average age of 83, five members were male (80%), and participants’ sedentary time ranged from 4 to 13 h per day.

**Table 1:** Characteristics of recruited participants

Participants	Gender	Age	Sedentary hours (weekday)	Sedentary hours (weekend)	Frailty Classification	Living Arrangements	Ethnicity
1	F	83	4	6	Mild	Lives Alone	White British
2	M	84	8	8	Severe	Lives Alone	White British
3	M	82	9	10	Moderate	Lives with Spouse	White British
4	M	82	10	10	Severe	Lives Alone	White British
5	M	84	13	13	Moderate	Lives with Spouse	White British
6	M	83	13	13	Mild	Lives Alone	White British
Average		83	9.5	10			
SD		0.89	3.4	2.8			

### Overview of findings

This analysis, informed by the ecological model of sedentary behaviour and the COM-B model of behaviour, identified the types of sedentary activities participants engaged in, and their perceived barriers and facilitators to reducing sedentary time. These frameworks aligned to the Behaviour Change Wheel used to guide the intervention development process [17], and provided insights into how sedentary behaviour in this population is shaped by individual, social and environmental influences. Several analytical themes emerged from this process, including shifting perceptions of sedentary behaviour throughout older adulthood, the impact of daytime sleeping on energy levels, and how social influences can promote or discourage sedentary lifestyles. These themes highlight the complex interrelationship between personal, social, and contextual factors that influence sedentary behaviour in older adults.

### Descriptive theme: activities performed in sitting

Sedentary activities, mostly leisurely and home-based, were mapped to the ecological model of sedentary behaviour [31]. The reported sedentary and non-sedentary activities are provided in Table 2.

**Table 2:** Activities reportedly performed in sitting and standing

Domain	Seated Activities	Non-Sitting Activities
Leisure	- TV	- Walking
	- Reading	- Shopping
	- Puzzles/Crosswords	- Gardening
	- Tablet/Computer use	- Meeting friends
	- Resting/Relaxing	- Socialising
	- Knitting/Sewing	
	- Playing games/cards	
	- Socialising with Friends	
Household	- Eating	- Making tea
	- Drinking	- Answering phone calls
	- Computer usage	- Getting dressed
	- Resting/Napping	- Chores/Tidying up
		- Getting medication
		- DIY
Transport	- Public transport (bus/train)	- Walking
	- Driving	- Walking to/from public transport
		- Cycling
Occupation	- Employment requires sitting	- Volunteering
	- Computer usage	- Helping friends/family
	- Teaching	
	- Attending Courses	

### Descriptive theme: barriers and facilitators to reducing sedentary behaviour

The barriers and facilitators to reducing sedentary behaviour were charted to the COM-B Model of behaviour change (Table 3) and are described narratively below.

**Table 3:** Participant responses charted to the COM-B model of behaviour

COM-B Domain	Barriers	Illustrative Quotes		Facilitators	Illustrative Quotes	
Physical Capability	Fatigue	“And that has caused me to cut down a lot on walking because I can’t walk well. But I can’t get far along there without have a breath and sit down.”	P5-84 M-MoF	Reduce Pain and Stiffness	“In fact, put it that way. So standing up, and sitting down themselves, absolutely mobilising the body, aren’t they?”	P3-82 M-MoF
	Physical Health	“Twice a week we go out walking. And then I started to have problems with one hip, which led to another hip. And that while we were ever going through that period, over two years, we relinquished our membership of the walking club, we never really got back to long distance walking, owing to the problems”	P4-82 M-SF	Manage Physical Health	“If you sat for a very long time and you stand up, you’d find yourself first. So that must indicate that the longer you sit down that the less beneficial it is for your body?”	P3-82 M-MoF
Psychological Capability	Depression	“If I’m a bit down, and I think I quite like to seek a very simple comfort of a chair”	P6-83 M-MF	Improve Sleep	“I can be asleep in literally any three, four or five, five minutes at the most.”	P6-83 M-MF
	Anxiety	“Those problems that create that anxiety, those are the ones that come in and stay”	P6-83 M-MF	Improved Satisfaction	“I’m always pleased when I get home that I’ve been in sort of part of a company of people.”	P1-83 F-MF
Physical Opportunity	Home	“So we’ve decided that that’s where we’re going to downsize. It’s popularly called, with all that goes with that, quite horrendous.”	P3-82 M-MoF	Engaged in Activities (Employment, Volunteering)	“Yeah, I’m involved in all sorts of do a food bank that’s on a volunteer at the food bank. I’ve been for about 10 years. I like to be busy.”	P1-83 F-MF
	Bad Weather	“It’s got a big say in how many, how many hours you’re going to sit because it’s pouring down and there’s nothing you can do outside. You’re not going to go anywhere.”	P1-83 F-MF	Home Enabling Reduction of Sedentary Behaviour	“I’m not the best gardener in the world. But I’ve loved climbing trees, trimming the trees, all those things were physical, physical efforts that and hopefully contributed to the well-being. But it all contributed to me being very busy. And I was very busy but doing nothing around the house.”	P3-82 M-MoF
	Financial Constraints	(Referring to local activities) “Cause it’s quite an expensive activity and they will be sat at home instead.”	P5-84 M-MoF	Neighbourhood Enabling Reduction of Sedentary Behaviour	It’s been resurfaced recently they’re all walking at it is a blessing. It’s perfectly flat that our seats all the way around.”	P6-83 M-MF
	Poor Public Transport	“You’ve got to walk embarrassingly. You’ve got at least some distance to walk to a bus, which is obviously an activity itself.”	P3-82 M-MoF	Public Transport	The bus to me is a lifesaver, it’s lovely from a social point of view, it breaks down barriers.	P6-83 M-MF
Social Opportunity	Loss of Role	“Well, since retiring. I would’ve said. And I retired at 60. I had a job where I was on their feet for the full eight hours of work and on an evening when the telly goes on, I’ll sit, sit read all evening and not move.”	P2-84 M-SF	Social Support: Family	When family visit, I would find myself sitting less because I would be doing a lot more for them.	P1-83 F-MF
	Social Isolation	“I do know people that do just sit and sort of look out the window because they don’t know what to do, they have no hobbies, so I don’t think that would be a good thing”	P1-83 F-MF	Social Support: Friends	If you were going out just for the evening, yeah, for to do whatever, you know, the theatre or just meet friends. Because you wouldn’t be sitting.	P1-83 F-MF
	Reduced Social Support Network	“I will say I understand that I’ve lost a lot of our friends.”	P4-82 M-SF	Neighbourhood Promotes Reduction of Sedentary Behaviour	“We’ve got these very fancy cafes, you know with foreign names, that are lovely, the street culture you know”	P6-83 M-MF
		“I’ve had a similar experience. I’ve been to three funerals this year already.”	P6-83 M-MF		(talking about local park) “So we have that that’s local, very local, that’s in our neighbourhood, and is safe and looked after. So that’s good.”	P4-82 M-SF
Reflective Motivation	Not Aware of Consequences of Sedentary Behaviour	“If you could prove to me that it is beneficial? And in what way would that be beneficial?”	P4-82 M-SF	Maintain Independence and Awareness of Health Benefits	(talking about following intervention)” But I would certainly if my doctor said I need to. Definitely. That’s for my health and well-being.”	P1-83 F-MF
	Unaware of Sedentary Behaviour	“I used to think that if I’m active during the day…that I’m doing well and to sit in the evening would be fine…but I’m now reconsidering that only because I’ve read about this.”	P1-83 F-MF		I used to think if I’m active during the day, which I am that I’m doing well. I’m now reconsidering that. Only because I’ve read about this. Sitting and it’s not good for you.	P1-83 F-MF
		“I didn’t really think about the amount of time I spent sitting until was asked about it”	P6-83 M-MF			
Automatic Motivation	Habitually Sedentary	“But I will have sat watching that television. Some nights, not every night, some nights, I will have something from six o’clock to 10 or 11. And I probably won’t move at all. Actually, I stay in the same seat that I’ve always sat in.	P4-82 M-SF	Habitually Non-Sedentary	“And I had a job with the engineering where I didn’t sit down from going in to coming out. It was active all the time. Now it’s gardening, cycling or walking.”	P2-84 M-SF
	Enjoy Activities in Sitting/Easier to Sit	“Things like reading a book or doing your knitting - getting up, that wouldn’t be fun, it would spoil the enjoyment.”	P1-83 F-MF	Activities of Daily Living	“I mean, to do the ironing I have to stand up and I do the vacuuming standing all these are really household chores. Dusting cooking, I’ve got to stand up if I wash the windows I have to stand up. Making the bed or mowing the lawn or do sweeping the path”	P1-83 F-MF

Key: *MF* Mild Frailty, *MoF* Moderate Frailty, *SF* Severe Frailty

#### Physical capability

Pain, fatigue and physical health problems contributed to participants’ sedentary behaviour. Participants perceived this decline as age-related and that their sedentary behaviour had increased throughout older adulthood. Members still performed non-sedentary activities but would perform them for shorter durations or replace them with less taxing activities. Some members recognised that prolonged sitting contributed to their pain and stiffness, and reducing sedentary behaviour may improve this.

#### Psychological capability

Members reported that their mental health, particularly feelings of depression and anxiety, increased their sedentary behaviour. When present, members described having little motivation to engage in physical activities, instead opting for sedentary endeavours. Members also described older adults that they knew who found it difficult to leave their homes for fears of falling or following the bereavement of a spouse. Conversely, members described how reducing their sedentary time, particularly through social activities, improved their overall mood.

#### Physical opportunity

Participants identified barriers to reducing sedentary behaviour at home and in the external environment. Home-related barriers included lack of space, (single-storey dwellings) and the implications of downsizing the home in later life. External barriers explored how public transport, neighbourhoods, poor weather, and financial constraints contributed to increased sedentary behaviour. Home-related facilitators included the presence of stairs, larger living areas and garden access can reduce sedentary behaviour. External facilitators which promoted the reduction of their sedentary time included employment or volunteering, affordable public transport, enticing and affordable local facilities, and outdoor seating in public areas.

#### Social opportunity

Participants described the impact of retirement on sedentary behaviour. For some members, their work was active, and upon retiring, they continued performing non-sedentary activities which they enjoyed that kept them active. Others described using volunteering to mitigate the loss of role following retirement, as it would provide the necessary structure and organisation to their day whilst discouraging sedentary behaviour. Although not applicable to the members, social isolation was described as promoting sedentary behaviour in older adults, with participants discussing older adults they knew who were socially isolated and would use sedentary activities to pass their day. Additionally, the impact of bereavement on social support networks and social opportunities was discussed. Social support from family and friends was an important facilitator in reducing their sedentary time.

#### Reflective motivation

Participants described how they did not consider their sedentary time, were largely unaware and unconvinced of the negative health consequences of their sedentary behaviour, nor the benefits of reducing their sedentary time. Some members were aware of the consequences of prolonged sedentary time and could reason that reducing their sedentary time may improve their health. Additionally, upon being presented with the evidence, participants were amenable to reducing their sedentary behaviour if it would improve their well-being.

#### Automatic motivation

Members expressed how established routines contribute to prolonged uninterrupted sitting, particularly in the evenings. These habits were firmly ingrained in their daily lives and oftentimes included sedentary activities which they enjoyed and had little interest in changing. Conversely, some members described being habitually active following employment in non-sedentary jobs. Necessary activities of daily living such as preparing food, drinking, and getting medication were motivators to interrupting their sedentary behaviour.

### Analytical themes

#### Theme 1: perspectives on sedentary behaviour throughout older adulthood

Participants’ perspectives on sedentary behaviour evolved throughout the discussions, which highlighted the complexity of its meaning and perceived implications across older adulthood. Initially, sedentary behaviour was often described in simple, or negative terms such as “sitting doing nothing” (P5-84 M-MoF). However, other participants introduced nuance by distinguishing between passive and mentally engaging sedentary activities, such as reading, sewing and knitting: *“To be sitting occupied. Yeah*,* reading something… I wouldn’t want to sit a long time. Just sitting. If I was sitting sewing*,* I’d have accomplished something”* (P1-83 F-MF).

Technology was a common point of reference in some participants’ conceptualisation of sedentary behaviour, being described as a contributor to prolonged sedentary behaviour: *“I have often heard it linked to modern technology and worries about by individuals who seem to experience it directly*,* as for being glued to the computer but perhaps it’s inevitable.” (*P6-83 M-MF). Technology was helpful in making group members more aware of their sedentary time, with two having experience with activity monitoring devices, however, the majority lacked awareness of their sedentary time: *“I didn’t really think about the amount of time I spent sitting until was asked about it” (P5-84 M-MoF).*

There was tension between participant’s awareness of their sedentary time, and their belief that being physically active during the day would counteract their sedentary behaviour: *“I used to think if I’m active during the day*,* which I am that I’m doing well. I’m now reconsidering that. Only because I’ve read about this. Sitting and it’s not good for you.”* (P1-83 F-MF). This view was predominantly held by group members who were more physically active, who weren’t convinced that sitting could be detrimental if otherwise active: “So I’d like to know how they come to the solution that is detrimental to anybody to sit?” (P4-82 M-SF).

Similarly, they questioned the health relevance of reducing sedentary time: *“If you could prove to me that it is beneficial? And in what way would that be beneficial?”* (P4-82 M-SF). Some members were aware of the health benefits of reducing their sitting time: “That must indicate that the longer you sit down that the less beneficial it is for your body?” (P3-82 M-Mod); whereas others were more amenable to change if supported by sources they deemed credible: *“I would certainly if my doctor said I need to. Definitely. That’s for my health and well-being”* (P1-83 F-MF). In the accompanying intervention development work, participants chose educational approaches delivered by credible sources (e.g. healthcare professionals) that addressed the distinction between physical inactivity, the health risks of prolonged sedentary behaviour and the benefits of reducing sedentary time.

Participants also believed that sedentary behaviour was a necessary component of their day during older adulthood: *“I think as you get older*,* it’d be difficult. What could you do to keep moving? Sensibly like*,* what would they want us to do? Besides just sitting?”* (P2-84 M-SF). They attributed this increase in sedentary time was a natural response to physical decline: *“My Dad used to say the hills are getting steeper. And as I get older*,* I realise he wasn’t daft at all. I was the daft one.”* (P6-83 M-MF). This decline resulted in them reducing the physical activities they enjoyed: “Twice a week we go out walking. And then I started to have problems with one hip, which led to another hip…over two years, we relinquished our membership of the *walking club*,* we never really got back to long distance walking”.* (P4-82 M-SF).

These physical limitations were compounded by age-related societal expectations: *“She also comes by public transport as though we both come in by helicopters. Does nobody travel by bus? As though it was something quite*,* you know*,* extraordinary*” (P1-83 F-MF). This quote illustrates the subtle social messaging that older adults should limit mobility or that remaining active is exceptional rather than normative. Additional perspectives on social influences on sedentary behaviour are provided later. This theme highlights how older adults’ perceptions of sedentary behaviour are influenced by their understanding, and are shaped by physical limitations, social norms and varying levels of understanding of the health consequences of sitting throughout older adulthood.

#### Theme 2: sleep and the energy balance

Although sedentary behaviour considers waking behaviours, participants described sleep and fatigue as key determinants of their movement and resting patterns. Fatigue, and disrupted sleep were described as normal features of ageing and framed as adaptive responses to changing energy demands and influenced their daily activity.

All participants reported experiencing daytime napping but had mixed perceptions of the activity. For some, it was something that was shameful or a source of embarrassment: *“I suppose this is a confession. I hope I’m not being too awful. I tend to have a nap in the afternoon… you lot don’t”* (P6-83 M-MF). Others viewed napping as an inevitable aspect of daily life: *“I don’t avoid them*,* I can’t avoid them necessarily*,* it happens!” *(P5-84 M-MoF)*. *One participant reframed this rest positively by adopting terms such as “power nap,” often validated by advice from peers or health professionals: *“I sometimes feel guilty over an afternoon nap. But a nurse friend of mine said don’t think that sleeping call it a power nap…”* (P6-83 M-MF).

Sleep-related fatigue was associated with a number of causes, including poor nighttime sleeping: *“I like to have a good night’s sleep I’m a really bad sleeper. It’d be nice if I woke up and felt refreshed”* (P6-83 M-MF); medication side effects: *“I have my breakfast*,* and I have my tablets and those tablets that I’ve got make you drowsy and I find I go off to sleep again having only got out of bed in an hour or so beforehand”* (P5-84 M-MoF); or evening tiredness: *“If I haven’t slept*,* I can sit down probably about half past five. And just nod off*,* you know*,* just go for about half an hour.”* (P4-82 M-SF). This tiredness oftentimes affected participants’ activity planning: *“I’ll sleep for half an hour or something like that. And then I feel awful because I wanted to get on or I wanted to go out” (*P1-83 F-MF).

Post-nap experiences also varied considerably among participants. Some described napping as akin to pacing strategies, and was used to revitalise them physically: *“When you’ve got a nap like that*,* even if it’s only for five minutes you find that that has revived you mentally physically for more than two-three hours”* (P4-82 M-SF), whereas others felt disorientated and sluggish: **“***For a good 10 minutes*,* I feel dreadful and don’t know where I am when I wake up.”* (P5-84 M-MoF). These accounts highlight how daytime napping can serve as a self-regulation strategy but may not always have positive outcomes, illustrating the importance of individual contexts in how sleep and fatigue influence sedentary behaviour. This suggests that both energy management and activity promotion should be considered when attempting to reduce sedentary behaviour in older adults.

#### Theme 3: sedentary behaviour and social connectedness

Throughout the series of focus groups, social connectedness emerged as a key influence on sedentary behaviour, potentially due to the group members being socially active. To participants, movement was seen as a means to facilitate social interaction, not just to meet a physical goal. Socially interacting with others can provide both motivation and opportunities for movement: *“We’re social animals. We need each other. And to be with other people you have to make the effort to move*,* which means moving and getting up and getting out of the chair at home”* (P6-83 M-MF).

The perceived benefits of social activity for healthy ageing were highlighted by participants: *“I think being in the company of other people*,* whether old or young*,* preferably*,* the whole mix*,* I think it keeps you young”* (P1-83 F-MF). Social interaction was described as a facilitator to reducing sedentary time and commonly arose through interactions with loved ones: *“I have two sons that are married. With kids. It’s like almost having three houses now. Because I do the decorating*,* and all three*,* painting and everything. So you have the opportunity.”* (P4-82 M-SF). Even if social activities were sedentary, participants could recognise how this reduced their sedentary behaviour: *“I’ll do a sewing group. But you see*,* we’re sitting down with that. But even with that*,* commuting across and getting there”* (P1-83 F-MF).

Conversely, the absence of social support was seen as a barrier to reducing sedentary, especially among those who lived alone: *“Yeah. Difficult. Because there’s nobody there to disturb you.”* (P1-83 F-MF), one participant replied when asked about the impact of living alone. Other barriers included reduced social support due to bereavements in their social network, which was reported by all participants and contributed to declining physical and social activity: **“***I think one of the worst things is that when you reach our age. You lose some of your close friends.”* (P4-82 M-SF). Participants noted that loneliness was increasingly common following the COVID-19 pandemic: *“What struck me was the number of people that said they felt so lonely”* (P6-83 M-MF). In the absence of this social interaction, older adults can readily find themselves becoming socially isolated: *“I do know people that do just sit and sort of look out the window because they don’t know what to do*,* they have no hobbies”* (P1-83 F-MF).

Although discussions were initially framed around sitting, by the end of the focus group series, participants began to recognise the broader psychosocial factors that influence sedentary behaviour: “Although this is on the surface, about physical acts, sitting down, actually it’s about the way we think and act, isn’t it?” (P6-83 M-MF). Participants did not view excessive sedentary behaviour as a purely physical issue, but instead, as an interaction between physical, social and mental well-being: *“We could separate social care*,* from physical and mental challenges*,* but we shouldn’t really separate them*,* because it’s all a part of us*,* in our minds and our bodies and our circumstances.”* (P3-82 M-MoF). Social contact, even when sedentary, provided both meaning and satisfaction to participants’ lives: “I’m always pleased when I get home that I’ve been in the company of people” (P1-83 F-MF). Social interaction was also described as beneficial for wellbeing, and a lens through which activities were experienced: “I think it’s, it’s quite apparent that being in other people’s company is really, really beneficial” (P6-83 M-MF). These findings highlight the interactions between social connectedness and sedentary behaviour in community-dwelling older adults, suggesting that promoting social engagement may be important for reducing sedentary behaviour in this population.

## Discussion

This study explored the perceptions of adults aged ≥ 75 years towards sedentary behaviour. Participants reported feeling like their sedentary behaviour progressed throughout older adulthood, and that their sedentary activities remained the same, but that they were performing them for longer periods similar to previous findings [32]. Descriptive themes focused on the barriers and facilitators to reducing their sedentary behaviour and the activities performed in sitting. Physical activities they deemed strenuous were replaced with less physically intense activities. Two descriptive themes and three analytical themes were also identified, each of which will be discussed below.

The ecological model and COM-B model of behaviour served as theoretical frameworks to guide the deductive thematic analysis to understand the behavioural determinants, settings and contextual factors that influence older adults’ decisions to sit [31, 33]. Sitting activities were predominantly leisurely in nature, and occurred in older adults’ homes. Interventions which have targeted non-specific reductions of sedentary behaviour observed the greatest reductions in television viewing and reading time [8, 34]. With certain sedentary activities such as socialising providing cognitive benefits to older adults [35], interventions targeting specific passive sedentary behaviours such as television viewing may be warranted. The COM-B provided an insight into the life pressures that influence an older adult’s decision to sit or stand. This study provides an insight into the dwindling capabilities, opportunities and motivations of older adults to reduce their levels of sedentary behaviour. In the wider literature, this gross multi-faceted decline is interconnected and almost cyclical in nature. Life transitions such as the bereavement of spouses and friends [11, 36–39], and the transition from employment to retirement [11, 37–40] predisposed to reduced opportunities to interrupt their sedentary behaviour. In turn, activity levels and physical functioning can decline as a result, making activities performed in standing more challenging, reducing motivation and promoting further sedentariness.

### Understanding of sedentary behaviour

Despite high self-reported levels of sedentary behaviour (8–13 h/day), participants did not view themselves as sedentary. This may be due to several factors. Firstly, as seen with McGowan and McEwan, there may be a reluctance associated with self-identifying as sedentary, as when asked to define sedentary behaviour were largely negative which may be attributed to a lack of understanding about sedentary behaviour [11, 36, 37]. Participants could define sedentary behaviour; however, these definitions were either negative or confused with physical inactivity. Secondly, members viewed sedentary behaviour as a harmless health behaviour and believed that being physically active during the day would mitigate prolonged periods of sedentary behaviour in the evenings. Participants were receptive to education as a strategy to address these misconceptions and reframe older adults’ perceptions of sedentary behaviour [17].

### Sleep

The increased frequency of daytime napping reported by participants may be attributable to age-related changes in circadian rhythm and sleeping patterns, cultural beliefs, chronic health conditions, medications, and lifestyle changes [41]. These associations have been observed in similar populations, such as stroke survivors [42]. Participants described the interrelationship between sleep and sedentary behaviour. A vicious cycle can be developed, where increased sedentary behaviour disrupts night-time sleeping [43] and disrupted night-time sleeping contributing to increased sedentary behaviour during waking hours [44]. In community-dwelling older adults, fragmented night-time sleeping is associated with a higher frequency of daytime napping [45], and an additional 26.5 min per day of sedentary screen time compared with normal sleepers (CI 5.2 min; 47.8 min) [46]. Furthermore, spending an additional 30 min per day sedentary is associated with poorer sleep quality [44], thus perpetuating the cycle.

Reducing sedentary behaviour in older adults has the potential to act as a non-pharmacological management option for disrupted night-time sleeping, as substituting 30 min of sedentary activity daily with equivalent durations of light-intensity physical activity correlates with improved sleep quality [47]. However, to some participants, daytime napping was described as almost akin to pacing strategies for fatigue management, with short periods of napping providing the necessary energy to complete their activities of daily living. Reviews of the literature suggest that shorter duration naps (< 30 min) are reported by older adults with better health, than longer duration naps (> 90 min), which are associated with poorer cardiovascular and diabetes outcomes, reduced cognitive function and increased mortality [41].

### Social connectedness

Participants highlighted the interconnection between social connectedness and sedentary behaviour in older adults. This topic is of growing importance as nearly half of adults aged ≥ 75 years live alone [48], and half of people aged ≥ 60 are at risk of social isolation and experiencing loneliness [49]. There is considerable overlap between the barriers to being socially connected and the barriers to reducing sedentary time, such as reduced physical well-being [50]. Contributing factors to this declining social connectedness included the impact of the bereavements of loved ones on social opportunities, with social network size decreasing with age [51]. Furthermore, significant associations have been identified between reduced social contact and sedentary behaviour in older adults [52]. Participants reported that social connectedness reduced their sedentary behaviour, whether through meeting friends, supporting family, or volunteering. Engagement in health-maintaining activities is associated with lower rates of social isolation [53]. Furthermore, social interaction may reduce sedentary behaviours linked to physical decline [54]. However, over 270,000 people in England aged ≥ 65 years have less than weekly contact with family or friends [55]. As sedentary behaviour increases, physical well-being and opportunities for social interaction diminish, creating a cycle of further isolation [56]. Further research is required to identify strategies to incorporate social support to reduce sedentary behaviour for older adults, particularly for those socially isolated.

### Strengths and limitations

Strengths of this study include that it recruited adults aged ≥ 75 years, an underrepresented subsection of older adults in sedentary behaviour research. Additionally, with all members belonging to the oldest old category, these focus groups offer novel insights into the perspectives of adults aged 80–85 towards sedentary behaviour throughout older adulthood. Furthermore, group discussions and the interactions between the participants may have yielded deeper conversations than may have been accomplished through semi-structured interviews. Additionally, the varying levels of sedentary behaviour, frailty status, and living arrangements of participants provided a wide range of perspectives.

The use of a single focus group across multiple sessions introduced methodological limitations, including the potential influences of group familiarity on the openness of discussions. As such, this may have limited the perspectives that were captured. However, this group familiarity provided continuity and facilitated trust, which was important for the co-production process to inform intervention development [17]. Additionally, the small number of participants recruited were socially active and were able and willing to attend group sessions. The ability to leave their home, travel and be comfortable sharing their views may preclude many older adults from participating, and their views may not be captured, particularly for older adults who are socially isolated. Participants were asked to consider other older adults in their lives when answering certain questions in an attempt to mitigate this. Furthermore, all participants were white British nationals, and this study was unable to identify any cultural influences that influence sedentary behaviour in this population. Targeted recruitment strategies are warranted to capture the views of the underrepresented subsections of the population in future research. Additionally, only one member of the focus groups was female, as two female participants withdrew before the study commenced due to familial matters. This gender imbalance may limit the generalisability of the findings given that women constitute the majority of people aged 75 years and above in the UK [57]. Another limitation of this work is that the socio-economic status of participants was not identified. All participants were recruited from Bradford, the 5th most income-deprived and 6th most employment-deprived local authority in England [58]. The impact of socio-economic deprivation on sedentary behaviour in community-dwelling older adults has previously been explored [11, 37], and overlaps in how older adults view sedentary behaviour and the influence of social connectedness on sedentary behaviour were identified. Additionally, eFI scores used to categorise frailty were drawn from the CARE75 + cohort and varied in recency. For some participants, the scores were more than 12 months old at the time of the first focus group, which may not fully reflect their current frailty status.

## Conclusions

This study suggests that while sedentary behaviour in adults aged ≥ 75 years shares similarities with younger older adults described in the wider literature, sitting activities are often reportedly sustained for longer periods of time, and barriers to reducing sedentary time occurring more frequently. This sample also demonstrated confusion between sedentary behaviour and physical inactivity, which may suggest that educational approaches may be helpful when attempting to reduce sedentary behaviour in this population. These findings provide insights into how sedentary behaviour may evolve throughout later life. However, given the small and demographically limited sample, further research is warranted to explore the perceptions in more diverse populations, particularly among more socially isolated older adults.

## Supplementary Information

Supplementary Material 1.

## Data Availability

The data that support the findings of this study are available from the corresponding author upon reasonable request.

## Declarations

All authors have read and approved the final manuscript.
