# Supplementary material for: Exploring the perceptions of sedentary behaviour in community-dwelling older adults aged 75 and older: a series of focus group interviews
Source: BMC Public Health. 2025 Jul 26;25:2558. doi: 10.1186/s12889-025-23793-y (PMC12297419; doi:10.1186/s12889-025-23793-y)
Supplement: Supplementary file 1 — Supplementary Material 1. [file 12889_2025_23793_MOESM1_ESM.docx]

Topic Guides for Qualitative Fieldwork

**Workshop 1 Interview Guide:**

- Introduction
- Rules
- Overview of session
- Video introduction

**Understanding of Sedentary Behaviour**

1. Had you heard the term “sedentary behaviour” before today, and if so could you describe it in your own words?
2. How would you describe your current levels of sedentary behaviour?
3. Are there any periods of day where you are more sedentary than others/less sedentary?
4. How has the amount you sit or lie down changed through the years? Has anything contributed to this?
5. How does sitting/lying down for long periods make you feel? Do you notice any effects?
6. How do you feel when you stand up? // What do you think the benefits of sitting less might be?

**Activities Performed in Sitting and Standing**

1. In front of you is a pen and notepad, I want everyone to take 5 minutes to write down as many activities and the reasons why you sit down throughout the day? – we’re going go around the group and make a big list of what we like to do.
2. We’re going to do a similar exercise, but we’re going to write down the things that the activities that are not in sitting.
3. How do you feel about incorporating more activity/moving around more into your day-to-day routine? **Have you taken up or tried to take up any new hobbies/activities?**
4. **Our final question deals with, we’re going to go around the table for this one, out of all the things we’ve talked about today, what to you is the most important thing that has been said – it might have been something you’ve said or something you heard someone else say - what do you think is most important – X can we start with you?**
5. **Did you have any final questions you wanted to ask this group – and if you don’t - would you give a little brief summary of the key points we talked about today**

**Recap**

1. **Did we capture that correctly? Is there anything that we missed or does that cover everything?**
2. **Dates – day and time preference – booking the room**

**Workshop 2 Interview Guide:**

| **COM-B Construct** | **COM-B Domain** | **TDF Domain** | Initial Question | Follow up/ Probing Questions |
| --- | --- | --- | --- | --- |
| **Capability** | **Psychological** | **Knowledge** | If a health care professional was to advise you to sit less, what do you think this might involve? | What do you think the benefits of sitting less might be? |
|  |  | **Memory, Attention, Decision Process** | What are the things that influence your decision to sit or stand? – follow up from the activities performed in sitting/standing | |
|  |  | **Behavioural Regulation** | Do you ever set any rules for yourself about when you should stand and move around? | |
|  | **Physical** | **Skills** | How easy or difficult would you find following that advice? | |
| **Opportunity** | **Social** | **Social Influences** | How do other people affect the amount of time you sit? | Anybody that helps or hinders reducing the time you sit? Perception of others? Groups? |
|  | **Physical** | **Environmental Context and Resources** | How does your environment affect the amount of time you sit? | Home – garden, stairs, space?  Neighbourhood – well maintained, safe, paths?  Travel – bus, train, car, cycle, walking?  Local facilities/clubs?  Time? Weather? |
| **Motivation** | **Reflective** | **Belief about Capabilities** | How confident are you that you would be able to reduce your sitting time?  How confident do you think these changes could be maintained?  What would help maintain these changes? | |
|  |  | **Belief about Consequences** | What might be the consequences of spending a lot of time sitting down?  How do you feel about these consequences?  What do you think will happen if you reduce the amount of time we sit? | |
|  |  | **Intention** | If an intervention was made, would you intend to follow it? | |
|  |  | **Goal** | To what extent would you follow it? | |
|  |  | **Optimism** | Do you think the barriers we’ve come up with today can be addressed/overcome? | |
|  | **Automatic** | **Emotion** | Does your sitting ever impact your mood/how you feel? | |
|  |  | **Reinforcement** | How would you incentivise sitting less during the day? | |
